# Glass-Forming Tendency of Molecular Liquids and the Strength of the Intermolecular Attractions

**DOI:** 10.1038/srep36934

**Published:** 2016-11-24

**Authors:** Kajetan Koperwas, Karolina Adrjanowicz, Zaneta Wojnarowska, Agnieszka Jedrzejowska, Justyna Knapik, Marian Paluch

**Affiliations:** 1 Institute of Physics, University of Silesia, ulica Uniwersytecka 4, 40-007 Katowice, Poland; 2 Silesian Center for Education and Interdisciplinary Research, ulica 75 Pulku Piechoty 1a, 41-500 Chorzow, Poland

## Abstract

When we cool down a liquid below the melting temperature, it can either crystallize or become supercooled, and then form a disordered solid called glass. Understanding what makes a liquid to crystallize readily in one case and form a stable glass in another is a fundamental problem in science and technology. Here we show that the crystallization/glass-forming tendencies of the molecular liquids might be correlated with the strength of the intermolecular attractions, as determined from the combined experimental and computer simulation studies. We use van der Waals bonded propylene carbonate and its less polar structural analog 3-methyl-cyclopentanone to show that the enhancement of the dipole-dipole forces brings about the better glass-forming ability of the sample when cooling from the melt. Our finding was rationalized by the mismatch between the optimal temperature range for the nucleation and crystal growth, as obtained for a modeled Lennard-Jones system with explicitly enhanced or weakened attractive part of the intermolecular 6–12 potential.

Any liquid which is progressively cooled down below the melting temperature will eventually solidify. This can happen in either of the two ways: by the formation of the crystalline solid of the regular arrangement of the molecules, or a disordered glassy state. Crystallization and glass-transition are two inherently related phenomena. Suppression of the crystallization plays a key role in the glass formation. Likewise, a liquid which cannot be supercooled ends up as a crystal. Controlling crystallization and its absence have a fundamental meaning in science and technology^1^,^2^,^3^,^4^,^5^. However, it is by no means easy to understand why some of the liquids are not prone to crystallize on cooling (even if a very slow cooling rate is applied), whereas undercooling of the other ones requires a tremendous effort^6^,^7^. On the other hand, some supercooled liquids which initially avoid crystallization on cooling from the melt become susceptible to crystallization when reheating from the glassy state^8^.

A set of guidelines for predicting the glass-forming ability of various liquids is dated back to Kauzman’s^9^, Turnbull’s^10^,^11^ and Uhlmann’s^7^,^12^ papers. For example, Kauzman noted that that glass-forming tendency is expected to improve with increasing *T*_*g*_*/T*_*m*_ ratio, where *T*_*g*_ is the glass transition temperature and *T_m_* the melting temperature. The value of *T*_*g*_*/T*_*m*_ ~2/3 is a lower-bound threshold for good glass-former. However, in many cases, the reduced temperature is not able to predict the glass-forming tendencies adequately (e.g. metallic or phosphate glasses^13^). Turnbull and Cohen found a correlation between the crystallization tendencies of simple liquids and the *T*_*b*_*/T*_*m*_ ratio where *T*_*b*_ is absolute boiling temperature of the liquid at atmospheric pressure. The condition for glass-formation requires *T*_*b*_*/T*_*m*_ to be near or more than 2. For molecular systems not crystallizing in the form of a small droplet 1.6 or more, while for those that clearly crystallize in small droplet form 1.5 or less. Early studies on the effect of molecular asymmetry on the glass-forming tendency have also indicated that asymmetric molecules, in general, can be supercooled easier than symmetric ones (famous pairs are toluene-benzene and p-xylene m-xylene)^6^,^11^. A set of these empirical rules has been later validated across series of molecules with different size, steric character or intermolecular interactions. Alba-Simionesco and co-workers have demonstrated that *T*_*b*_*/T*_*m*_ rule fails to predict good glass-forming tendencies of meta- isomers of benzene ring compounds with the hydrogen bonding substituents^14^. Wang and Richert have demonstrated a positive correlation between *T*_*g*_ and *T*_*b*_ for liquids that belong to various chemical classes. However, this can completely break for isomers^15^. Studies across homologous series of tris-napthylbenzene^16^ and cyclic stilbenes^17^ have also pointed out that the variation of the melting point (even by 5%) might give a significant impact on the glass-forming tendency. Based on the experimental observations, understanding the glass-forming tendency of various liquids seems to be a very challenging task.

Looking for the source of the different crystallization behavior of the molecular liquids intuitively leads to the important role of the intermolecular interactions, similarly as they affect numerous of the structural and dynamics features of the viscous liquids^18^,^19^,^20^. The evidence for that can be given from the experimental study, e.g. when the crystallization is avoided by changing the hydrogen bonding propensity, or the molecular composition of the studied samples^21^,^22^,^23^. Even for colloidal suspensions, the phase behavior and crystallization kinetics can be changed by adding polymers that produce attractions between the particles^24^,^25^,^26^.

In the field of the molecular dynamics simulation, much of the research efforts concentrate on studying the effect changes in the pair-wise potential on the glass transition and crystallization phenomena^18^,^19^,^27^,^28^,^29^,^30^. However, systematic studies on the role of the attractive (or repulsive) forces on the tendency to crystallize/vitrify are rare^31^,^32^,^33^,^34^. We mean here in particular systems consisting of the particles of the same kind, interacting with each other in an isotropic and non-selective manner. In consequence, the essence of the intermolecular forces in determining the stability of various materials against crystallization remains a puzzle. The multi-component systems are indeed more explored. For example, Toxvaerd *et al.* demonstrated that binary Lennard-Jones mixture composed of molecules of A- and B- types with removed AA and BB pair attractions the system is faster to simulate and less prone to crystallize than the standard Kob-Andersen system^35^. However, identifying the fundamental role of the intermolecular interactions on glass-formation in multicomponent mixtures is more complex because of tortuous phase diagrams or necessity to define the interactions between particles of the different types. From this point of view, simple molecular liquids seem to be a better choice. Therefore, the motivation for this study is to identify at the most fundamental level the effect of changes in the interparticle interactions on the crystallization and glass-forming tendencies.

In this work, we focus specifically on the role of the attractive interactions which together with the repulsive forces determine the total intermolecular potential of the molecular liquids. We show that the tendency to crystallize of van der Waals liquids correlates with in the strength of the intermolecular attractions. The experimental attempt aimed to tune the attractive term was achieved by varying with the magnitude of the dipole-dipole interactions while keeping some other components to remain almost invariable. The choice of the investigated samples has a critical meaning, because crystallization/glass-forming tendencies might be affected by many factors. Focusing on the strength of attractive forces requires separating contributions coming from the different effects since they may also contribute to the glass-forming ability. In such case, finding substances of a given type with almost equally competent structure (or molecular properties) that differ predominantly in the magnitude of the dipolar interactions was a major challenge. This paper further presents the molecular dynamics simulations of a simple Lennard-Jones (LJ) liquid with explicitly enhanced (by a factor of 1.2) or weakened (by a factor of 0.8) attractive forces. Carefully designed experimental work combined with the computational results will lead to a remarkable conclusion that the stability of van der Waals liquids against crystallization correlates with the strength of the intermolecular attractions. In this way, by changing the magnitude of the attractive part of the intermolecular potential, we can indeed modify the crystallization and glass-forming behavior of the molecular liquids.

## Results and Discussion

### Description of the molecular liquids selected for the experimental study – factors expected to predispose glass-forming tendency

**Figure 1:**
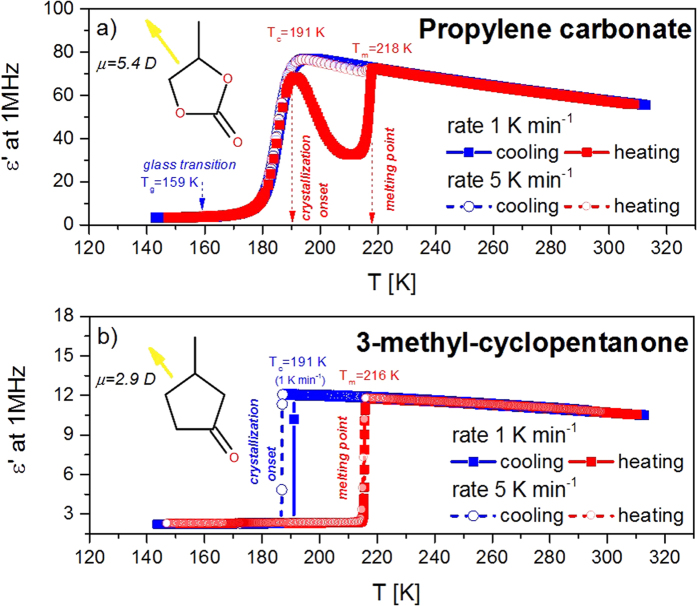
Evolution of the dielectric permittivity upon cooling and heating for propylene carbonate and 3-methyl-cyclopentanone. Temperature dependence of the dielectric constant *ε*′ for (**a**) propylene carbonate and (**b**) 3-methyl-cyclopentanone as measured at 1 MHz frequency upon cooling and heating with the rates 1 K·min^−1^ and 5 K·min^−1^. The evolution of *ε′* on cooling for propylene carbonate shows typical signatures characteristic for a good glass-forming liquid. However, a slight drop of *ε*′ as due to recrystallization is observed when heating the glassy sample. A sudden drop of the dielectric permittivity when cooling 3-methyl-cyclopentanone denotes crystallization event, whereas its abrupt increase on heating signifies the melting temperature. The insets show chemical structures of the investigated samples with the most probable orientation of the permanent dipole moment.

The experiments were performed on van der Waals liquids, propylene carbonate (PC) (MW = 102.08 g·mol^−1^) and 3-methylcyclopentanone (MW = 98.14 g·mol^−1^). Both are cyclic structural analogs of each other, as demonstrated in the insets of Fig. 1a,b. In 3-methylcyclopentanone, the two oxygen heteroatoms in the five-membered ring are replaced by the carbon atoms. This substitution does not have a significant impact on the molecular weight and the molecular architecture of the studied compounds. However, it affects very clearly the polarity of both molecules. Propylene carbonate is highly polar liquid with a large value of the dipole moment *μ* (4.94 D^36^ or even 5.36 D^37^, while 3-methylcyclopentanone is its less polar analogs. By applying the density functional theory (DFT), we obtain that the value of the dipole moment for 3-methylo-cyclopentanone is *μ *= 2.9 D, while *μ *= 5.4 D for PC. We also wish to note that such modification of the chemical structure affects only the magnitude of the dipole moment, but it does not change the orientation of the dipole moment concerning the molecular axis of the studied samples. Therefore, we were able to exclude additional anisotropy effects. The values and the directions of the dipole moments for PC and 3-methylcyclopentanone are presented in the Supplementary Fig. 1.

By applying the simple criteria predicting the glass-forming ability in molecular systems (mentioned in the introduction), we get that for both considered compounds they are almost equally competent. The melting points of PC (*T*_*m*_ = 217.5–218 K) and 3-methylcyclopentanone (*T*_*m *_= 216 K) varies less than 1%. Both molecules have the same steric character and molecular architecture. The *T*_*b*_*/T*_*m*_ ratio predicts the ease of glass-forming ability for both substances as obtained values are close to 2 (*T*_*b*_*/T*_*m*_ = 1.94 for 3-methylcyclopentanone and 2.35 for PC^38^). Hence, their fluidities at the melting points are expected to be not so much different, same as their tendency to form a glass. Interestingly, in both cases, the contribution coming from the thermodynamic driving force is also expected to be comparable. This is because the Gibbs free energy difference between the liquid and crystal states defined by Hoffman’s equation 
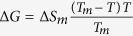
 produces almost equivalent results with Δ*S*_*m *_= 44 J·mol^−1^K^−1^ for PC and Δ*S*_*m *_= 48 J·mol^−1^K^−1^ for 3-methylcyclopentanone. The melting entropy Δ*S*_*m*_ can be obtained from the following relationship 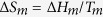 where Δ*H*_*m*_ is the melting enthalpy of the crystal (Δ*H*_*m *_= 9.62 kJ/mol for PC^39^ and Δ*H*_*m *_= 10.32 kJ/mol for 3-methylcyclopentanone). From the above consideration, we conclude that the classical principles describing the glass-forming tendencies of the molecular systems do not predict significant discrepancy between the glass-forming behavior of both samples selected for the present study. However, this contradicts with the experimental results as will be shown in the next part of the paper. When those most lasting rules fail, recalling to the fundamental aspect of the difference in the dipolar attractions can rationalize experimental observations.

### Impact of the dipolar attractions on the tendency to vitrify, as probed experimentally

To investigate the crystallization tendencies of both samples we have used dielectric and calorimetric techniques. In agreement with the principal idea of the dielectric measurements, fluctuations of the dipoles in the electric field are needed to get the dielectric response of the sample. The higher the value of the permanent dipole moment of the molecule, the better the dielectric response of a given material. We can express this by using dielectric permittivity. Propylene carbonate is known to be a highly polar van der Waals liquid, as reflected by the value of dielectric constant, *ε*′* *= 66 at 293 K^34^,^40^, It also shows an excellent glass-forming ability which together with its simple molecular structure makes it an interesting material to investigate the dynamic features of the viscous liquids near the glass-transition^41^,^42^,^43^,^44^,^45^. Indeed, when cooling down from room temperature, it is impossible to induce crystallization, even if a relatively low cooling rate is applied (1 K min^−1^). In Fig. 1a we illustrate changes in the dielectric constant *ε′* at 1 MHz frequency. As the temperature decreases, the behavior of *ε*′ for PC resembles that characteristic for a typical glass-forming liquid. This includes a systematic increase of the dielectric constant with cooling and the dielectric dispersion signatures. However, on heating the glassy sample with the rate of 1 K min^−1^ crystallization event was surprisingly detected at the temperature range around 30 K above the glass-transition temperature, *T*_*g *_= 159 K. The evidence for that is a sudden drop of the dielectric permittivity from the value *ε*′ ≅ 68 to approx. 30. The increase in the heating rate to 5 K min^−1^ makes the crystallization process to be hardly detectable.

In contrast to PC, 3-methylcyclopentanone reveals an entirely different stability behavior on cooling. As demonstrated in Fig. 1b, lowering the temperature always results in the crystallization, indicated by a sudden drop of the dielectric constant. Vitrification of 3-methylcyclopentanone was even not observed when applying the fastest accessible by us cooling rate, i.e. 50 K min^−1^. Therefore, we have classified it as very poor glass-forming liquid. Interestingly, for both investigated samples the melting point and the onset of the crystallization are located at approximately the same temperature region. The results of the complementary calorimetric studies are demonstrated in Supplementary Figs 2 and 3. Additionally, we have also constructed for the investigated samples CCT (Continuous-Cooling-Transformation) and CHT (Continuous-Heating-Transformation) diagrams, which are well known by the metallurgy community. CCT and CHT are a very useful way of analyzing the non-isothermal processes when the different transformation products are obtained depending on the cooling/heating rate. The CCT and CHT curves for PC and 3-methylcyclopentanone are presented in Supplementary Figs 4 and 5.

Intuitively, the difference in the polarity has come to the fore, when trying to explain the entirely different glass-forming tendencies of propylene carbonate and 3-methylcyclopentanone. Polar molecules attract each other via dipole-dipole interactions, which intensity is directly proportional to the magnitude of the permanent dipole moment. By taking this into account, it is clear that there should be a significant difference in the strength of the dipole-dipole attractions for both investigated samples. This idea corresponds to approximately 6-folds lower value of the dielectric permittivity for 3-methylcyclopentanone (*ε′* = 11 at 293 K) than PC, as obtained from the dielectric studies.

For the polar molecules unable to participate in the hydrogen bond formation, the dipole-dipole forces together with the induced-dipole-induced-dipole (dispersion) interactions determine the average intermolecular attractions. In the case of van der Waals bonded liquid having a permanent dipole moment *μ*, the total attractive interaction energy between the rotating molecules of the same type can be approximated as


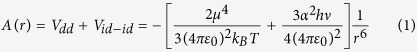


where *V*_*dd*_ and *V*_*id−id*_ describe the average dipole-dipole and induced-dipole-induced-dipole energies, respectively^46^. In Eq. 1 *ε*_*0*_ denotes permittivity of the vacuum, *α* is polarizability of the molecule, *hν* is the energy of the harmonic oscillator, and *r* is a distance between the molecules. The dispersion forces are the default intermolecular attractions resulted from the presence of the transient dipoles in all type of the molecules, both polar and non-polar. So, together with the dipole-dipole interactions they give rise to the total intermolecular attractions of the polar molecular liquid.

It is well-known that the molecular weight and the molecular size translates into the strength of the dispersion forces^47^,^48^,^49^. The samples selected for present study (PC and 3-methylcyclopentanone) have almost identical MW and the molecular architecture. On that basis, we can expect very similar contribution coming from the dispersion forces. So, indeed, the differences in the strengths of the dipole-dipole attractions could have a decisive impact on the magnitude of the overall intermolecular attractive forces. This is more evident when fixing all parameters in Eq. 1 except for the different values of the permanent dipole moment for PC and 3-methylcyclopenanone that changes to the fourth power. The corresponding intermolecular attraction energy for PC is expected to be eight times higher than for 3-methylcyclopenanone. Using DFT method we obtain theoretical values of the isotropic polarizability *α *= 67 [a.u.] for 3-methylo-cyclopentanole and *α *= 50 [a.u.] for propylene carbonate. The difference in the polarizability of both samples could not even produce a two-fold change because it changes to the second power in Eq. 1.

### Crystallization of the pure Lennard-Jones fluid with modified attractions forces

Up to this point, the experimental evidence has indicated that the changes in the attractive part of the intermolecular interaction potential correlate with the crystallization capability of the molecular van der Waals bonded liquids. Stronger attractions as due to the enhanced dipole-dipole interactions produce a good glass-forming ability of PC, whereas weakening of the intermolecular attractions (by lowering the contribution of the dipole-dipole forces) makes 3-methylcyclopenanone crystallize readily on cooling. However, to verify the hypothesis that the strength of the attractive forces influences on the tendency to crystallize/vitrify a more robust evidence and detailed understanding of this finding need to be provided. For that purpose, we have performed molecular dynamics simulations of the modeled system composed of the particles interacting through the Lennard-Jones (LJ) potential with explicitly strengthened or weakened the attractive part.

The total potential energy of the standard Lennard-Jones system *V*_*ST*_(*r*) separated by the distance *r* is defined as the sum of two terms, the short-range repulsions *R*_*ST*_ and the long-range attractions *A*_*ST*_^50^


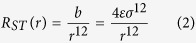


and


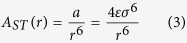


where *ε* and *σ* define the potential well minimum and the finite distance at which the potential energy is zero, respectively. To show the effect of the attractive forces on the crystallization properties of the LJ fluid, we have modified the strength of the attractive part, while keeping the repulsive term to be constant. This was achieved by introducing a control parameter *λ*


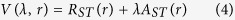


**Figure 2:**
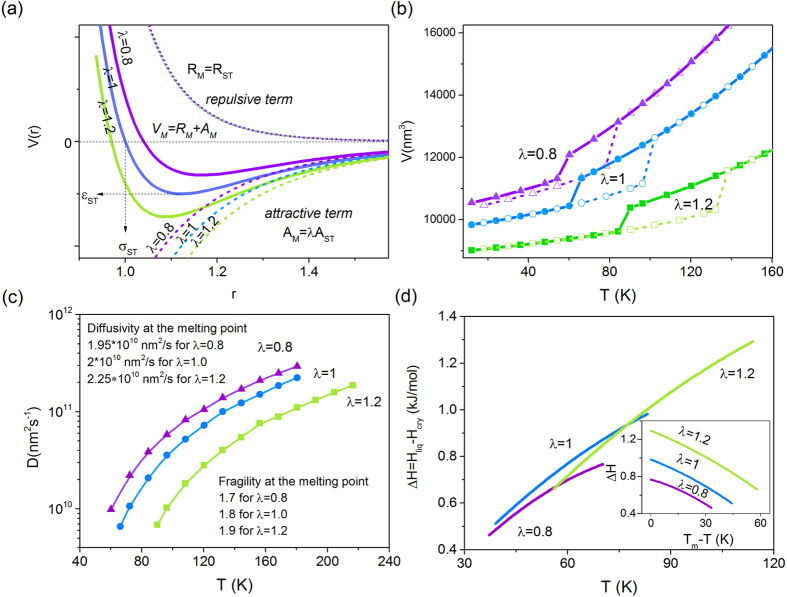
Thermodynamic and dynamic properties of the Lennard-Jones system with enhanced and weakened attractive part of the intermolecular potential. (**a**) The LJ potential with varying attractiveness used in this study. (**b**) Temperature evolution of the simulated system’s volume for liquid and crystal phases. The dashed lines refer to heating runs, while the solid to the cooling runs. (**c**) The diffusion coefficient and (**d**) the difference in the enthalpies between liquid and crystal phases plotted as a function of temperature for studied LJ system with *λ*-dependent attractive part of the intermolecular potential. The inset in panel (**d**) shows changes of Δ*H* versus *T*_*m*_*-T*.

In consequence, for the modified LJ system, we kept *R*_*M*_(*r*) *= R*_*ST*_(*r*), whereas the attractive forces were tuned by the *λ* parameter, *A*_*M*_(*r*)* = λA*_*ST*_(*r*). Similar procedure was also utilized by Hummer and co-workers to model carbon-water interactions in the nanotubes^51^,^52^. We have considered three cases with the parameter *λ *= 0.8, 1.0 and 1.2, where the increase of *λ* signifies the increase of the attraction strength. The resulted *λ*-dependent intermolecular potentials we demonstrate in Fig. 2a. We use GROMACS software to perform molecular dynamics simulations of 10648 same molecules interacting via LJ potential (as given by Eq. 4) at pressure p = 1.6 MPa and different temperatures. We describe simulations details in Methods Section.

The standard LJ model system is well-known for its simplicity and for its ability to readily crystallize. Nevertheless, it can reproduce surprisingly well some of the basic properties of the simple fluids. Therefore, from MD simulations, we have directly obtained the most important physical quantities for the studied LJ system such as density, melting temperature, diffusion coefficient or enthalpy. We present the results in Fig. 2b–d. One may notice that the physical properties of even such very simple modeled fluid depend strongly on the strength of the intermolecular attractions. The increase of *λ* parameter brings about a decrease in the volume of the liquid and the crystal phases. Stronger attractions also shift the melting point towards higher temperatures and causes a larger hysteresis on cooling and heating. From Fig. 2b we can also see that for the LJ system with weakened attractions the volume of the crystalline phase formed upon cooling does not match that as recorded upon heating. This result indicates that the solid phase formed upon cooling is less ordered, possibly as due to defects that accompany the process of crystal formation when quenching the liquid.

The strength of the attractive forces also affects the dynamic and thermodynamic properties of the LJ system. The diffusion coefficient *D* decreases with increasing *λ*, as can be seen in Fig. 2c. The diffusivity of the studied systems at the melting point is very similar, but one can also note a slight (and systematic) increase of D with increasing *λ.* Apart from that, LJ system with strengthening attractions seems to be a bit more fragile than that with depleted attractive term. The difference in the enthalpies between the liquid and crystal phases Δ*H* is expected to increase with increasing *λ*. The latter effect is evident when plotting versus *T*_*m*_*-T* (see inset in Fig. 2d). A similar evolution of the volume, the diffusion coefficient, and the enthalpy difference was reported for a one-component LJ system with tunable attractive part of the intermolecular potential as portrayed by the rational fraction^31^.

In the next step, the crystallization behavior of the investigated system with the modified strength of the intermolecular attractions was described in terms of the equations provided by the classical theory of nucleation and growth^53^,^54^,^55^. Here, we wish to note that the classical approach rests on numerous of the assumptions, and in many cases, it fails to provide the exact temperature dependences of the nucleation and growth rates. However, without doubts, it is considered as a fundamental guiding picture of the crystal formation^56^,^57^. Therefore, it was employed in this study to predict a possible nucleation and growth tendencies of the LJ fluid with the different attraction strength. Possibility to achieve a quite reasonable agreement between the nucleation times predicted from the classical nucleation theory and that obtained directly from MD simulations was demonstrated recently^31^.

**Figure 3:**
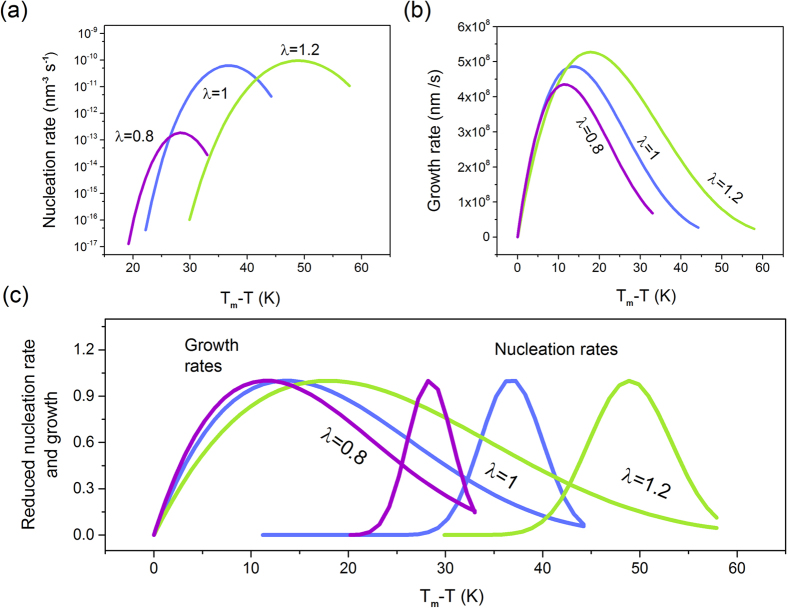
Location of the maxima of nucleation and growth for LJ system with the modified strength of the intermolecular attractive forces. Predicted by the classical approach evolution of the (**a**) nucleation and (**b**) growth curves for investigated LJ system with the λ-dependent strength of the attractive part of the intermolecular potential. (**c**) Normalized by the respective maximum values nucleation and crystal growth rates dependences plotted as a function of the undercooling.

The location and magnitude of the maximum rates were calculated using the physical quantities (such as diffusion, density or enthalpy) extracted directly from the MD simulations. In Fig. 3a,b we show the estimated nucleation and crystal growth curves for a LJ system with the different contribution of the attractive forces. To facilitate comparison, we plot obtained temperature dependencies as a function of the undercooling (*T*_*m*_*-T*). As can be seen, the increase of *λ* shifts the nucleation and growth rates maxima towards lower temperatures, away from the respective melting points. The predicted curves seem to be also broader, and of the greater magnitude. This could be an indication that the strengthening of the attractive component of the intermolecular potential promotes the nucleation and crystal growth process. However, regardless of the magnitudes of the nucleation and growth rates, their relative location is essential in determining the propensity of the liquid to vitrify or crystallize on cooling. Since the scales of the nucleation and growth rates cannot be directly compared, we have employed the reduced coordinates to analyze only the location of their maxima with respect to each other. For considered LJ system with different values of the *λ* parameter, we demonstrate that in Fig. 3c. Interestingly, with increasing the strength of the attractions the nucleation zone occurs at a much lower temperature than the growth area. In consequence, the optimal temperature ranges for nucleation and crystal growth become progressively separated from each other. Keeping in mind that the crystallization requires first nuclei to be formed and then its subsequent growth, we see that a liquid with stronger attractions can potentially bypass crystallization and form a glass on cooling. However, such system is also prone to crystallize on heating from the glassy state, after passing the nucleation and growth zones. In contrast, weakening attractive forces promotes crystallization of the LJ liquid on cooling as due to the temperature overlap of the nucleation and growth rates curves.

## Conclusions

We have demonstrated that the tendency of van der Waals liquid to vitrify or crystallize when cooling from the melt correlates with the strength of the intermolecular attractions. Selecting appropriate molecular analogs differing only in the strength of the dipolar interactions enable us to reveal this stringing finding very clearly in the experiment. While, by applying predictions of the classical theories of the nucleation and crystal growth to LJ fluid with a tunable attractive part of the intermolecular potential we get additional evidence that could explain it. The results indicate that the strength of the attractive forces affects crystallization behavior of the molecular liquids by changing the location of the nucleation and crystal growth rate maxima with respect to each other and the melting point. Therefore, van der Waals liquids with stronger attractions are expected to supercool much easier, as their optimal temperature range for nucleation and growth shifts apart. In contrast, weakening attraction forces favor crystallization on cooling because both maxima are in proximity to the melting point. We envisage that this finding is potentially important to understand the fundamental problem of what makes a liquid be a good (or bad) glass-former, especially that for many samples it is impossible to explain the differences in the glass forming tendency by resting only on the well-established criteria known from Uhlman’s and Turnbull’s papers.

Understanding the nature of glass-forming ability is also a very significant problem for designing metallic glasses and their usage in engineering applications. In many metallic glasses, it is impossible to predict their glass-forming ability by using the frequently used criteria and that the glass-forming ability varies in a non-linear way with concentrations of atoms in the alloy. Our finding that the crystallization tendency of the molecular liquids increases with enhancing the strength of the attractive forces is quite similar to the evidence provided by Sha and co-workers for Cu-Zr alloys^58^,^59^. In this case, the same effect was achieved by improving the electronic stability of the basic cluster as due to stronger bonding of atoms in the cluster that originates from the strong coupling of their s, p and d electrons. This knowledge can be possibly used to tune the crystallization behavior of liquids by manipulating directly at the most fundamental level of the intermolecular interactions. Our future research will focus on studying the effect of the anisotropy of the interparticle interactions, pressure effects and the contribution of the short range repulsions in determining the crystallization tendencies of the molecular systems.

## Methods

### Materials

Investigated liquids propylene carbonate (*M*_*W *_= 102.09 g mol^−1^) and 3-methylcyclopentanone (*M*_*W *_= 98.14 g mol^−1^) were of purity >99% were purchased from Sigma-Aldrich and used as received.

### Dielectric Measurements

Dielectric permittivity was measured with the use of a Novocontrol GMBH Alpha dielectric spectrometer. The temperature was controlled by Quattro Novocontrol system with a temperature stability better than 0.1 K. The liquid samples were placed between two stainless steel electrodes separated by a gap of 0.1 mm provided by a quartz ring. Measurements of the dielectric permittivity at 1 MHz frequency were performed at the various rates under a nitrogen atmosphere.

### Calorimetric Measurements

Calorimetric measurements were carried out by Mettler-Toledo DSC apparatus equipped with a liquid nitrogen cooling accessory and a HSS8 ceramic sensor (heat flux sensor with 120 thermocouples). Temperature and enthalpy calibrations were performed by using indium and zinc standards. The samples (approx. 12 mg) were sealed in aluminum pans. Aluminum pans with samples have been top sealed with one puncture. Standard DSC runs were performed at the various rates under a nitrogen atmosphere.

### Quantum Mechanic Computations

Theoretical studies were carried out within the framework of density functional theory (DFT) in the ORCA package (program version 3.0.03)^60^. Calculations were performed on isolated molecules. In the first step, we have performed geometry optimization at B3LYP/6-31 G** level. The SCF was converged tightly (TightSCF, Energy change 1.0e-08 au). DFT integration Grid2 (Lebedev 110 points and IntAcc = 4.34) was used for SCF Iterations and Grid4 (Lebedev 302 points and IntAcc = 4.67) for the final energy evaluation after SCF convergence. Then, the values of the dipole moment and polarizability tensor were calculated on optimized structures at B3LYP level and using 6-311 G** basis set. The static (frequency independent) polarizability was calculated analytically through the solution of the coupled-perturbed CP-SCF equations for DFT run and expressed in atomic units (a.u.) which are widely used in theoretical atomic physics. The conversion to Å^3^ unit requires multiplying by 0.1481847 factor. Calculating molecular properties were performed within TightSCF, Grid5 and FinalGrid6 tolerance. For more details, please see ORCA manual (https://orcaforum.cec.mpg.de/OrcaManual.pdf).

### Simulation Details

We have used the GROMACS software to perform standard simulations of the molecular dynamics at constant temperature and pressure controlled respectively by the Nose-Hoover thermostat and Martyna-Tuckerman-Tobias-Klein barostat. The systems under investigations contained 10648 identical molecules, built from one atom of the one unified atomic mass unit, located in a cubic cell with the periodic boundary conditions. The interactions between molecules were described by the Lennard-Jones potential, as given by Eq. 4. For standard Lennard-Jones liquid the attractive and the repulsive interactions were expressed respectively as 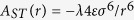 and 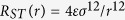 with 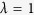, 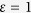 and 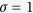. For the modified systems, we kept the repulsive term constant, 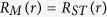, whereas the attractive part was strengthened or weakened by choosing an appropriate value of the *λ* parameter. For stronger attractions we set 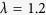, so that the attractive part of the intermolecular potential can be given as 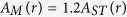. This corresponds to the following values of 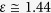 and 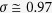 In the case of the system with the weakened attractions 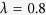, and the attractive part of the intermolecular potential was defined as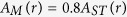. This results in 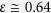 and 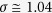 The potential was truncated at a cut-off distance 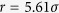 i.e. *5r*_*min*_ where *r*_*min*_ is the position of the minimum of the potential. The energy 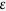 and the length 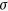 units of the modeled LJ system were expressed using GROMACS basic units^61^, i.e. *nm* is a basic unit of the length, whereas energy is specified by *kJ/mol*. Consequently, *ε* and *σ* are equal to 1 *kJ/mol* and 1 *nm* respectively for the standard LJ system (*λ *= 1). The potential parameters do not reproduce the physical properties of the molecular liquids used in the experiment. However, we want to stress that our goal is to study systems which differ only in the strength of attractive interactions. And to minimize the complexity of that problem we have chosen very simple LJ fluids.

The single-component Lennard-Jones systems are well-known to crystallize very easy. Crystallization can be detected by analyzing the temperature dependence of the volume at constant pressure. To obtain the above dependence, we have performed a series of the molecular dynamics simulations at pressure, *p *= 1.6 MPa, and different temperatures. We have used the velocity Verlet algorithm with time step 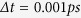 for integrating Newton’s equation of motion. The starting point was in an equilibrated liquid state at *T *= 180 K. The temperature was decreased from 180 K down to 12 K with a temperature step ΔT =6 K. Then, it has been subsequently increased again to the starting point. During this experiment, we have observed crystallization and melting of the systems, respectively. Each simulation run was performed for minimum 200000 time-steps. If the volume of the system kept constant value (with a very good approximation) during last 100000 time-steps, we recognized that the system is in an equilibrium. And, for those time-steps, we have collected the values of the volume, enthalpy, and the diffusion. In the vicinity of the crystallization and the melting points we have increased the number of the time-steps for equilibration ten times. For each system, we have described the temperature dependence of the volume in the liquid and the solid states using the quadratic equation. The hysteresis between the temperature dependence of the volume for cooling and heating runs was observed^62^. Therefore, we have estimated the melting temperature according to the equation, 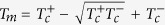 where 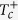 and 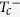 are maximum degrees of superheating and supercooling^63^.

### Prediction of the nucleation and crystal growth rate curves

We have described the possible crystallization behavior of the investigated LJ system as expected from the classical theory. For that purpose, the physical quantities extracted from the MD simulations were used (such as density of the liquid and crystal phases, melting temperature, enthalpy, diffusion coefficient). The location and magnitude of the nucleation and growth rate curves were calculated using equations provided by the classical theory of nucleation and growth. The steady state rate of nucleation for a spherical nucleus was expressed as^64^,^65^


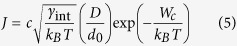


where *c* is the particle number density, *d*_0_ is characteristic size parameter 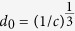, 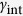 is the specific interfacial energy, *D* is the effective diffusion coefficient and W_c_ is the thermodynamic barrier to nucleation expressed as^66^,^67^


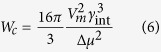


In the above equation *Vm* is the molar volume of the crystallizing phase and Δ*μ* is the difference in the chemical potential per particle of the liquid and crystalline phases acting as a fundamental thermodynamic driving force towards crystallization. Here, we calculate Δ*μ* using the equation proposed by Gutzow *et al.* (Eq. 6 in ref. 59) with the redefined integration pathway (in an analogous way as done in ref. 68). Since for all examined systems we consider identical isobaric conditions, the pressure corresponding to it was defined by us as a reference pressure, and then the equation for driving force proposed by Gutzow takes the following form


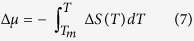


where Δ*S* is the difference in the entropy between its values for liquid and crystal phases. We have estimated Δ*S* using obtained directly from the simulation runs dependences *H(T*) for a liquid and crystal (which were approximated by the polynomial equations), and the relation between the enthalpy and the entropy, 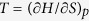 (taking into account the boundary condition 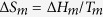). The temperature evolution of the thermodynamic driving force towards crystallizations for the studied LJ system is presented in Supplementary Fig. 6.

The crystal*–*liquid interfacial free energy *γ*_int_ was calculated according to the formula suggested in ref. 59 and 60 which at isobaric conditions corresponding to the reference pressure can be rewritten,


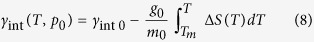


where parameters g_0_ and *m*_0_ are defined in the same way as proposed by Gutzow and coworkers^59^. The value of *γ*_int0_ for the LJ system considered was estimated using Scapski-Turnbull formula 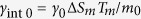 where 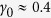. In Supplementary Fig. 7 we present the obtained temperature dependence of *γ*_in*t*_. Herein, we wish to note that the liquid/solid interfacial free energy for the studied LJ systems is subtle in comparison to that reported for ‘real’ materials. However, our simulated systems are much simpler. So, the values of their liquid/solid interfacial free energy can be indeed much smaller.

Using estimated temperature dependences of Δ*μ* and *γ*_in*t*_, we have calculated the nucleation rate, *J*, and crystal growth rate, *U*, in the way predicted by the classical theory. To calculate the growth rate, we have used the following expression^69^,^70^


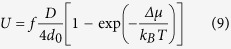


where 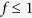 is a parameter that has different values, depending on the growth mechanism. In the simplest model, i.e., normal growth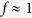. For homogeneous nucleation the value of *f *= 1 and less than 1 when homogeneous nucleation cannot be guaranteed. The diffusion coefficient was estimated from the long-term evolution of the mean square displacement calculated at different temperatures in the liquid state. For temperatures lower than the melting temperature *D* was obtained by approximation of the *D(T)* dependence for liquid by Vogel-Fulcher-Tammann equation^71^,^72^,^73^.

## Additional Information

**How to cite this article**: Koperwas, K. *et al.* Glass-Forming Tendency of Molecular Liquids and the Strength of the Intermolecular Attractions. *Sci. Rep.* **6**, 36934; doi: 10.1038/srep36934 (2016).

**Publisher’s note:** Springer Nature remains neutral with regard to jurisdictional claims in published maps and institutional affiliations.

## Supplementary Material

Supplementary Information
