# Supplementary material for: Glass-Forming Tendency of Molecular Liquids and the Strength of the Intermolecular Attractions
Source: Sci Rep. 2016 Nov 24;6:36934. doi: 10.1038/srep36934 (PMC5121653; doi:10.1038/srep36934)
Supplement: Supplementary Information [file srep36934-s1.pdf]

# Supplementary Materials

## Glass-Forming Tendency of Molecular Liquids and the Strength of the Intermolecular Attractions

Kajetan Koperwas[1,2], Karolina Adrjanowicz[1,2*], Zaneta Wojnarowska[1,2], Agnieszka Jedrzejowska[1,2], Justyna Knapik[1,2] & Marian Paluch[1,2]

[1] *Institute of Physics, University of Silesia, ulica Uniwersytecka 4, 40-007 Katowice, Poland*

[2] *Silesian Center for Education and Interdisciplinary Research, ulica 75 Pulku Piechoty 1a, 41-500 Chorzow, Poland*

**Supplementary Figures**

(a) (b)

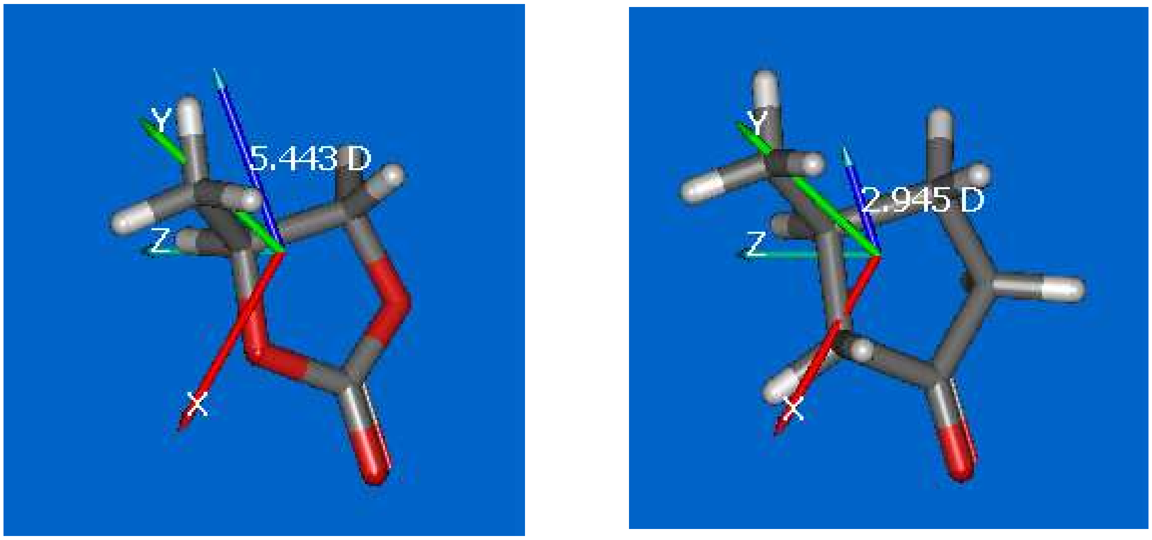


**Supplementary Figure 1. Orientation of the dipole moment.** Optimized geometry of (a) PC and (b) 3-methyl-cyclopentanone. Blue arrows indicate orientation of dipole moment with respect to principal inertial axes.

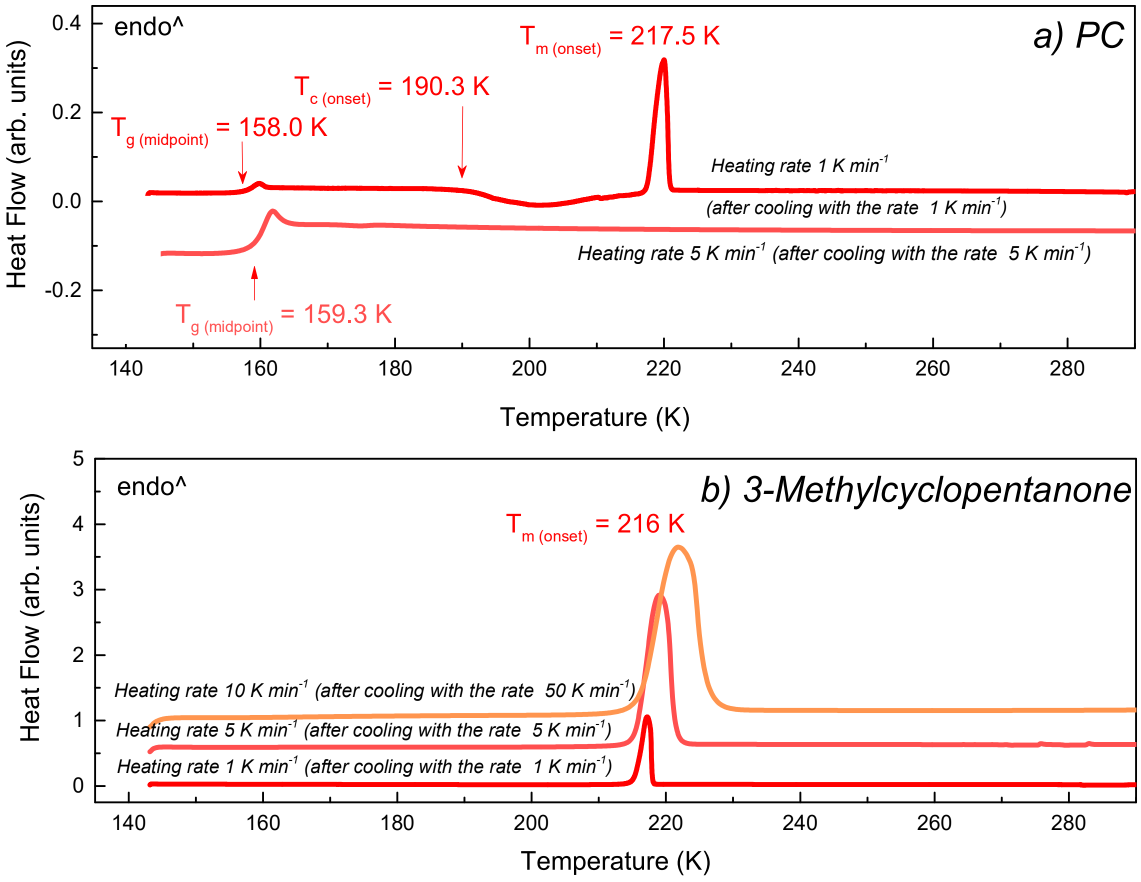


**Supplementary Figure 2. Results of the calorimetric measurements**. DSC thermograms for (a) PC and (b) 3-Methylocyclopentanone recorded on heating with different rates. No glass transition event was detected in DSC of 3-methylcyclopentanone.

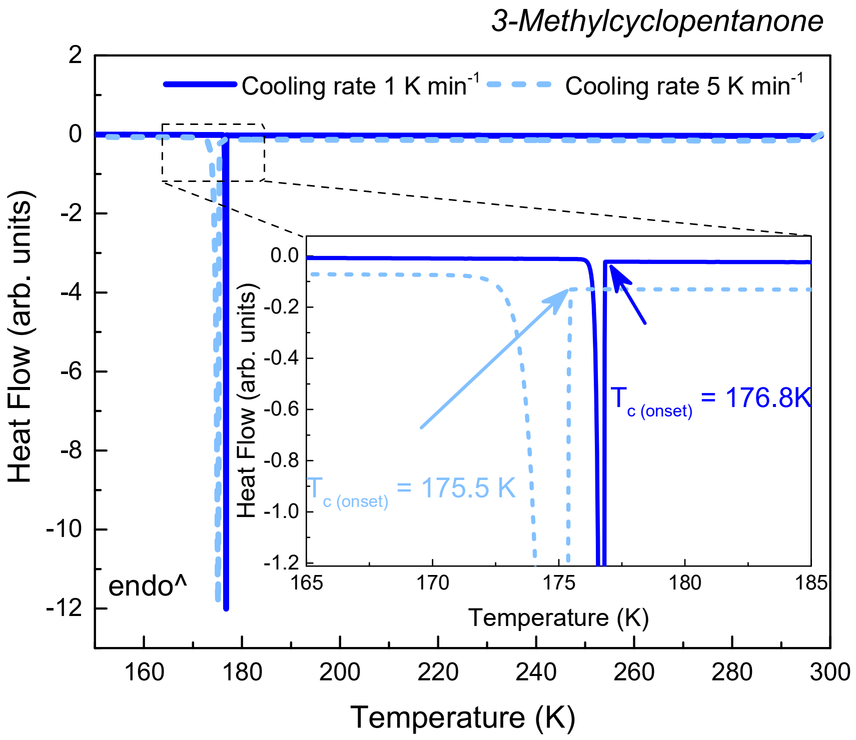


**Supplementary Figure 3. Results of the calorimetric measurements**. DSC thermograms recorded for 3-Methylcyclopentanone on cooling with different rates.

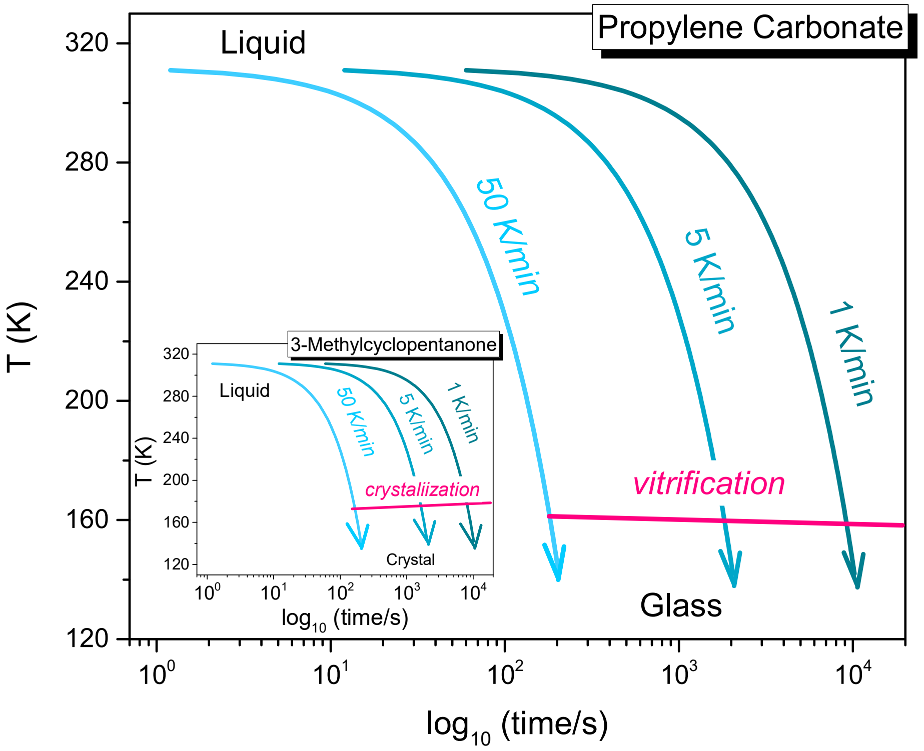


**Supplementary Figure 4.** CCT curve for PC and 3-methylcyclopentanone (inset).

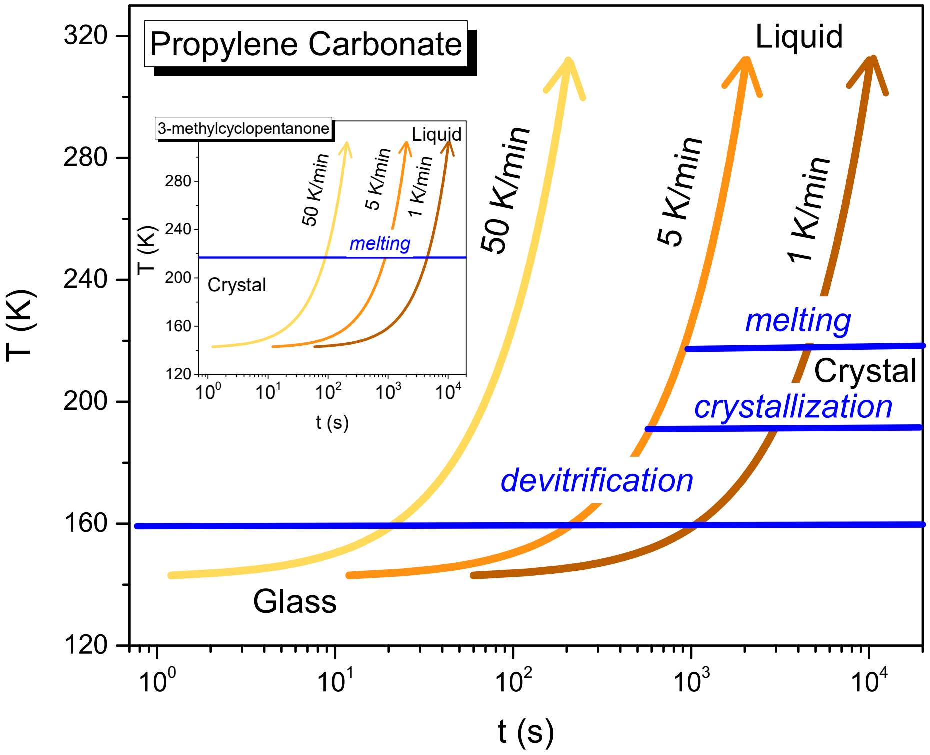


**Supplementary Figure 5.** CHT curve for PC and 3-methylcyclopentanone (inset).

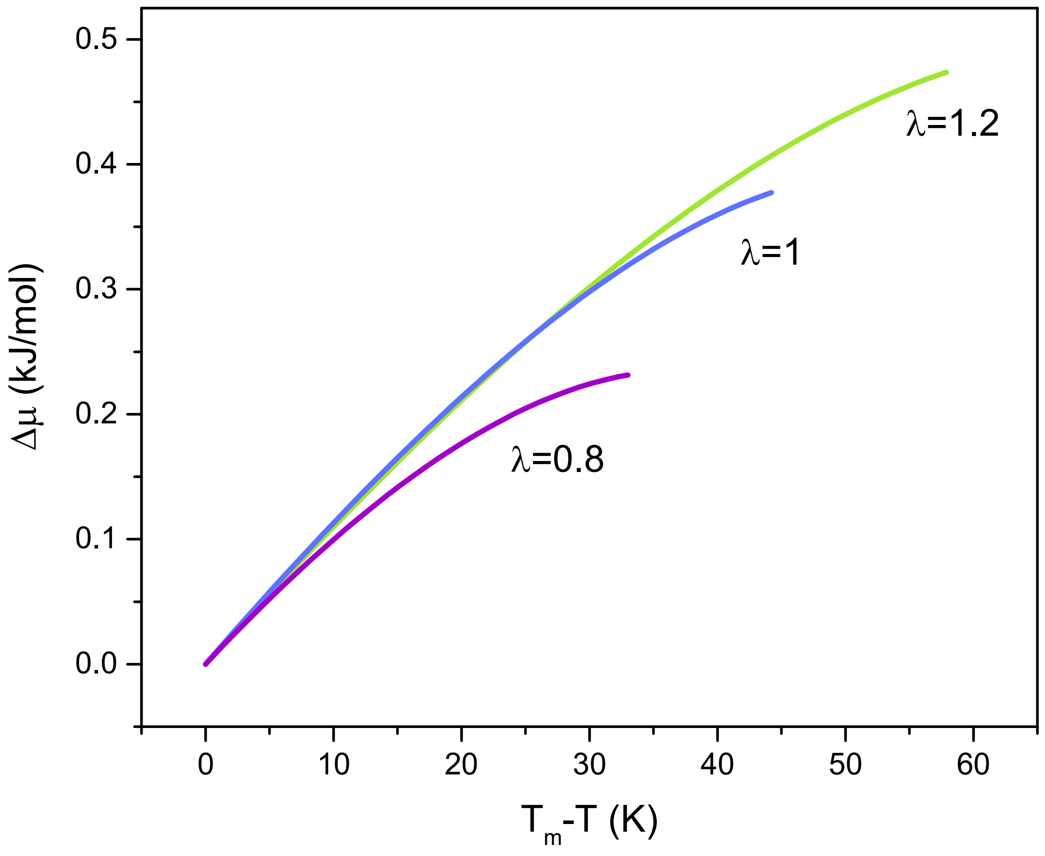


**Supplementary Figure 6. Temperature evolution of the thermodynamic driving force towards crystallization**. Possible temperature behavior of $\Delta\mu$ for LJ system with varying strength of the intermolecular attractive forces, predicted using Eq. 3.

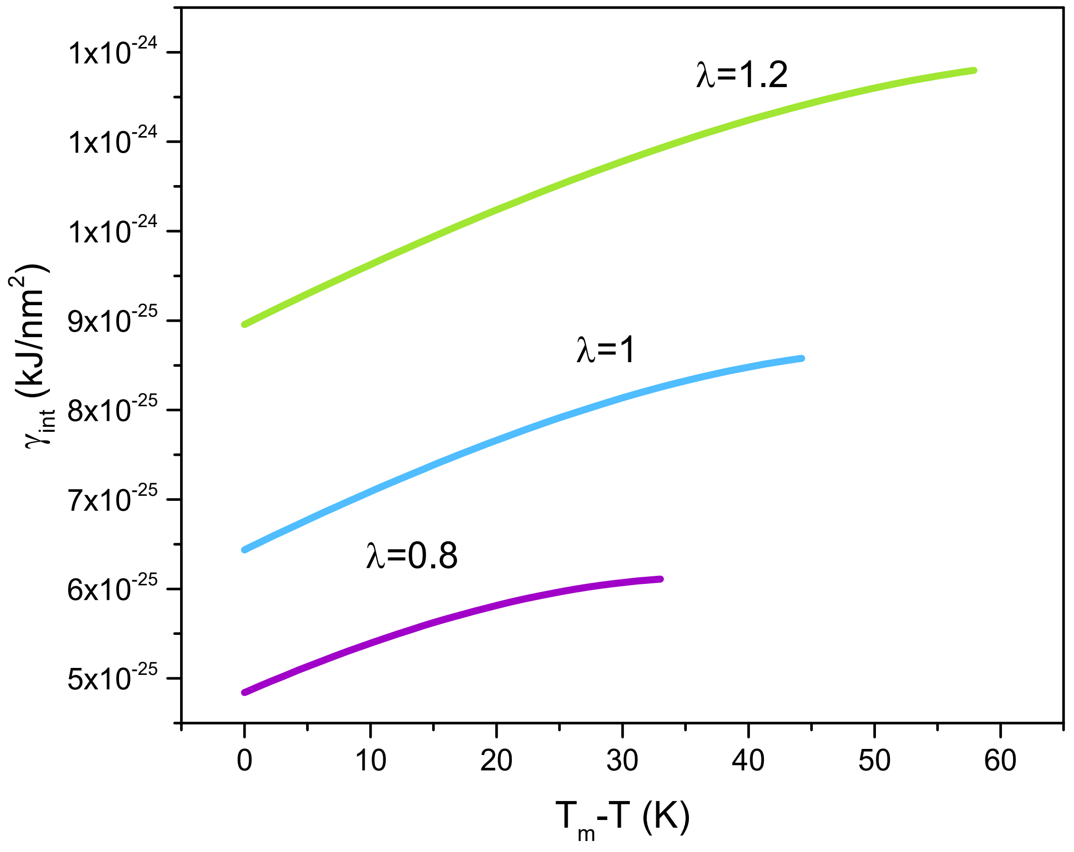


**Supplementary Figure 7. Temperature evolution of the specific surface energy**. Possible temperature behavior of $\gamma_{int}$ for LJ system with varying strength of the intermolecular attractive forces, predicted using Eq. 4.
